# New iodine-apatites: synthesis and crystal structure

**DOI:** 10.3906/kim-2102-5

**Published:** 2021-10-19

**Authors:** Evgeny N. BULANOV, Sergey S. PETROV, Alexander V. KNYAZEV

**Affiliations:** 1 Department of Analytical and Medicinal Chemistry, Faculty of Chemistry, Lobachevsky University, Nizhny Novgorod Russia

**Keywords:** Iodine, apatite, synthesis, crystal structure, Rietveld refinement

## Abstract

The paper describes methods for the preparation of compounds with an apatite structure containing only iodine atoms in the “halogen” position. The crystal structure of the compounds was refined by the Rietveld method. The resulting apatites have a structure with a space group *P*6_3_/*m* and have the following unit cell parameters: Ba^4^ *^f^*_1.78(2)_Ba^6^ *^h^*_2.75(2)_(PO_4_)_3_I_0.04(2)_ (*a* = 10.18609(34) Å, *c* = 7.71113(30) Å, *V* = 692.889(54) Å^3^, *R* = 5.448 %), Pb^4^ *^f^*_1.82(2)_Pb^6^ *^h^*_2.75(2)_(PO_4_)_3_I_0.13(2)_ (*a* = 9.87882(18) Å, *c* = 7.43222(16) Å, *V* = 628.144(26) Å^3^, *R* = 8.533 %), Pb^4^ *^f^*_1.90(2)_Pb^6^ *^h^*_2.68(2)_(PO_4_)_3_I_0.16(2)_ (*a* = 9.87058(48) Å, *c* = 7.41255(46) Å, *V* = 625.437(72) Å^3^, *R* = 5.433 %). The study of the crystal structure showed a relatively low efficiency of the binding of iodine in the apatite matrix.

## 1. Introduction

Currently, 37 isotopes of iodine are known. Among them, the greatest attention is paid to iodine-129: it is one of the seven long-lived fission products of uranium and plutonium and in significant quantities entered the atmosphere as a result of nuclear tests in the 1950s and 1960s [1]. At the same time, it also poses a danger to humans, as a more rapidly decaying iodine-131, since, due to its nature, it can accumulate in the body.

To bind various isotopes of iodine, including iodine-129, many approaches have been proposed [2, 3]:

• mercurex-process, implying receipt of its mercury compounds,

• iodox-process, allowing to fix iodine in the form of solid precipitate HI_3_O_8_,

• use of sorbents based on titanium oxide, copper, silver.

In a number of works [4–6], it was proposed to use a matrix with the structure of the mineral apatite for binding isotopes of iodine, in particular, long-lived iodine-129 (T*_1/2_* = 1.57(4)⋅10^7^ years).

The structural type of apatite is known for its high isomorphic capacity, due to which it can undergo substitutions in the structure by almost all atoms of the periodic table and within wide quantitative limits [7, 8]. It should also be noted that, unlike the structural types traditionally used as the basis of matrices for binding radionuclides (garnet, pervoskite, hollandite, monazite, etc. [9, 10]), apatites are able to bind not only cations but also anions; therefore, the attention directed at them in the context of radioactive iodine binding is quite justified.

The general formula of apatites can be described as follows: M^4^ *^f^*_2_M^6^ *^h^*_3_(AO_4_)_3_L, where M stands for cations of different oxidation states, located in two crystallographically different positions of the structure, A refers to the most often atoms prone to the formation of tetrahedral coordination polyhedra (for example, P, V, Si, S), and L is for halogens or OH-groups, as well as O^2-^, CO_3_^2-^ and other ions. The content of cations in the structure is rather high; therefore, geoceramics, including apatites, are studied to bind strontium-90 [11, 12]. Taking into account the crystal-chemical similarity of apatite to the mineral part of the native bone of mammals, similar processes can be observed in the human body: at this limit of strontium accumulation in bone tissue, thermodynamic modeling is possible [13].

Attempts to theoretically predict the possibility of binding iodine by the apatite structure were undertaken earlier [14], but a more detailed result was obtained in [15], where, based on machine learning, density functional theory, DFT (density functional theory), and experimental data 18 new thermodynamically stable compounds with the apatite structure containing iodine anions were predicted. Since the attention to the issue of the efficiency of the binding of iodine by the apatite matrix does not decrease [16, 17], an attempt is made in this work to partially reproduce and expand the results of theoretical modeling.

## 2. Materials and methods

### 2.1. Synthesis

To obtain iodine-apatites, two approaches were considered, namely wet and solid-state approaches.

In the solution method of synthesis, 0.5 M solutions of nitrate of the corresponding divalent cation, ammonium hydrogen phosphate, and a solution containing a 2-fold excess of potassium iodide were used. The general scheme of the reaction can be represented as follows:

5M^II^(NO_3_)_2_∙nH_2_O + 3(NH_4_)_2_HPO_4_ + KI → **M** **^II^** **_5_** **(PO** **_4_** **)** **_3_** **I** + 6NH_4_NO_3_ + KNO_3_ + 3HNO_3_ + 5nH_2_O (1)

The resulting precipitates were kept in the mother liquor for a day, then centrifuged with rinsing with bidistilled hot water and dried in air.

Solid-phase synthesis implied the preparation of a stoichiometric mixture of ammonium hydrogen phosphate, as well as nitrate and iodide of a divalent cation. The reaction mixture was calcined sequentially at temperatures of 300°C, 500°C, and 800°C. The calcination time was 4 h at each stage with the dispersion of the mixture in an agate mortar during the transition to each next stage.

4.5M^II^(NO_3_)_2_·nH_2_O + 0.5M^II^I_2_·mH_2_O + 3(NH_4_)_2_HPO_4_ → **M** **^II^** **_5_** **(PO** **_4_** **)** **_3_** **I** + 9NO_2_ + 2.25O_2_ + 6NH_3_ + (4.5 + 4.5n + 0.5m)H_2_O (2)

Both approaches are simpler in terms of hardware design than the microwave synthesis proposed in [18], the mechanochemically activated method in [19], or the synthesis using electro-pulse plasma sintering in [4]. The particular amounts of used compounds are given in Table 1. 

**Table 1:** Amounts of compounds used in two synthetic approaches.

Wet synthesis				
Target compoundcomposition	V(M(NO3)2∙4H2O, 0.5 M)(ml)	V((NH4)2HPO4, 0.5 M)(ml)	V(KI, 0.5 M)(ml)	m (apatite)(g)
Ca5(PO4)3I	42.3	25.4	16.9	not apatite
Sr5(PO4)3I	47.3	28.4	18.9	not apatite
Ba5(PO4)3I	38.3	23.0	15.3	not apatite
Cd5(PO4)3I	32.4	19.5	13.0	not apatite
Pb5(PO4)3I	30.2	18.1	12.1	3.5974
Solid-state synthesis				
Target compoundcomposition	m(M(NO3)2∙4H2O)(g)	m((NH4)2HPO4)(g)	m (MI2∙nH2O)(g)	m (apatite)(g)
Ca5(PO4)3I	5.0000	1.6776	1.2445	not apatite
Sr5(PO4)3I	5.0000	1.8720	1.6133	not apatite
Ba5(PO4)3I	5.0000	1.5159	1.6345	4.11911
Cd5(PO4)3I	5.0000	1.2843	1.1872	not apatite
Pb5(PO4)3I	5.0000	1.1961	1.3919	3.69362

1with barium phosphate (comments in text).2after losing of the greater part of PbI2 (comments in text).

We used reagents manufactured by the Vekton company of analytical grade and chemically pure grade, except for lead (II) iodide, which was synthesized by the solution method by the reaction of saturated solutions of lead nitrate and potassium iodide and characterized by X-ray diffraction.

### 2.2. Research methods

The phase individuality of the obtained compounds was monitored using a Shimadzu XRD 6000 powder diffractometer. Powder X-ray diffraction patterns were taken in the 2θ angle range of 10–60 °, on an X-ray tube with a copper cathode (λ (CuKα) = 1.5406 Å) at a voltage of 30 kV and a current 30mA.

Chemical purity and composition of the obtained sample were studied with Shimadzu XRF-1800 spectrometer using fundamental parameters (FP) method with using standard example. BaK_α_, CaK_α_, PbK_α_, IK_α_, PK_α_ lines intensities were measured three times at 40 kV, 50 mA on Rh anode with FPC detector for P, and SC detector for Ca,Ba,Pb,I (Table 1). Investigation of the chemical composition of the samples was also performed on method energy dispersive X-ray microanalysis (EDXMA) with Oxford Instruments X-MaxN 20 detector.

To refine the crystal structure, the method of full-profile X-ray analysis (the Rietveld method) was used [20]. The X-ray diffraction patterns were taken on the same diffractometer in the 2θ angle range of 10–120°, the X-ray tube voltage of 40 kV and the current strength of 40 mA, the exposure at a point was 11 s. The structures of known apatites with large halogens Sr_5_(PO_4_)_3_Br [21], Cd_5_(VO_4_)_3_I [22], and Pb_5_(VO_4_)_3_I [19] were considered as primary models. The pseudo-Voight function (PV_TCHZ) was used to describe the peak profile. The crystal structure was refined using the Topas 3.0 software package.

To estimate the particle size, we used both the data on the refinement of the crystal structure of the Topas 3.0 program and calculations using the Scherrer formula:

(3)d=kλβ cosθ

where *d* is the average crystal size, *K* is the dimensionless particle shape factor (Scherrer’s constant, for spherical particles is considered equal to 0.9), *λ* is the wavelength of X-ray radiation, *β* is the width of the reflection at half height, θ is the diffraction angle [23].

Microscopic studies by high-resolution transmission electron microscopy were performed on a JOEL JEM2100F transmission microscope at a voltage of 200 kV, and by atomic scanning microscopy on an AURIGA CrossBeam Workstation (Carl Zeiss).

## 3. Results

In this work, an attempt was made to obtain iodide-trisphosphates of a number of divalent cations (Ca, Sr, Ba, Cd, Pb) with an apatite structure.

X-ray phase analysis of precipitates obtained in the course of solution synthesis, as well as polycrystalline samples obtained by the solid-phase method, showed that, in the overwhelming majority of cases, orthophosphates of the corresponding divalent cations were obtained. The exceptions were solution synthesis with lead (hereinafter PbPI (w)) and solid-phase syntheses with barium and lead (BaPI and PbPI (ss), respectively): their diffraction patterns were similar to the X-ray diffraction patterns of compounds with apatite structure presented in the inorganic crystal structure database (ICSD).

X-ray fluorescence analysis showed (Table 2) that the amount of bound iodine in the resulting precipitates is significantly less than expected from the theoretical stoichiometry of the compounds (especially in the case of barium compound).

**Table 2:** X-ray fluorescence analysis data for synthesized1 compounds.

Target compoundcomposition	BaO (wt%)			P2O5 (wt%)			BaI2 (wt%)		
	calc	found2	found3	calc	found2	found3	calc	found2	found3
Ba5(PO4)3I	62.82	75.76	75.74	19.38	23.38	23.42	17.80	0.86	0.84
	PbO (wt%)			P2O5 (wt%)			PbI2 (wt%)		
	calc	found2	found3	calc	found2	found3	calc	found2	found3
Pb5(PO4)3I	69.37			14.71			15.92		
solid-state		80.56	80.54		17.08	17.08		2.36	2.38
wet		80.02	80.02		16.96	17.00		3.02	2.98

1Standard uncertainties u are u(wt) = 0.02%.2Shimadzu XRF-1800.3JEOL JSM-IT300LV with Oxford Instruments X-MaxN 20 detector.

**Figure 1:**
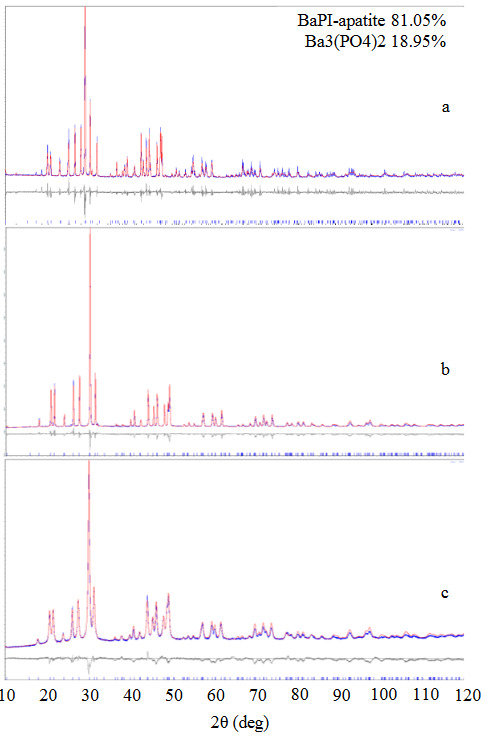
Experimental (blue), calculated (red), difference (gray), and stroke X-ray diffraction patterns of the obtained compounds: BaPI - a, PbPI (ss) - b, PbPI (w) - c.

A full-profile X-ray analysis of the obtained compounds showed that their crystal structure corresponds to the type of apatite with the space group *P*6_3_/*m* of the hexagonal system. In addition, the quantitative phase analysis of the obtained samples showed that the BaPI sample contains a significant impurity of barium phosphate, the structure of which was taken from [24]. The phase analysis of the lead-containing samples showed the absence of any secondary phases in the final product (Figure 1a–1c, Table 3). In the case of PbPI-ss, it can be explained by the absorption of most of the melt of lead iodide by the material of the alundum crucible (T_m_ (PbI_2_) = 412°C).

**Table 3:** Parameters and results of full-profile X-ray analysis of the crystal structures of the synthesized iodine-apatites.

	BaPI	PbPI(ss)	PbPI(w)
Space group	P63/m		
a (Å)	10.18609(34)	9.87882(18)	9.87058(48)
c (Å)	7.71113(30)	7.43222(16)	7.41255(46)
V (Å3)	692.889(54)	628.144(26)	625.437(72)
М (g·mol-1)	910.7(96)	1246.7(60)	1255.0(81)
Crystal size (nm)	331(64)	203.1(85)	27.58(63)
Density (g·cm-3)	4.365(23)	6.591(16)	6.664(21)
Coefficients of pseudo-Voight function			
U	1.014(55)	–0.205(10)	0.133(38)
V	–0.7411(25)	0.1424(72)	0.008(32)
Q	0.1300(14)	–0.0229(12)	–0.0187(66)
Z	0	0	0
X	0.000(12)	0.0542(56)	0.000(17)
Y	0	0	0
Scale factor	0.00013737(86)	0.0003990(11)	0.00013517(44)
R-Bragg (%)	5.448	8.533	5.433

**Figure 2:**
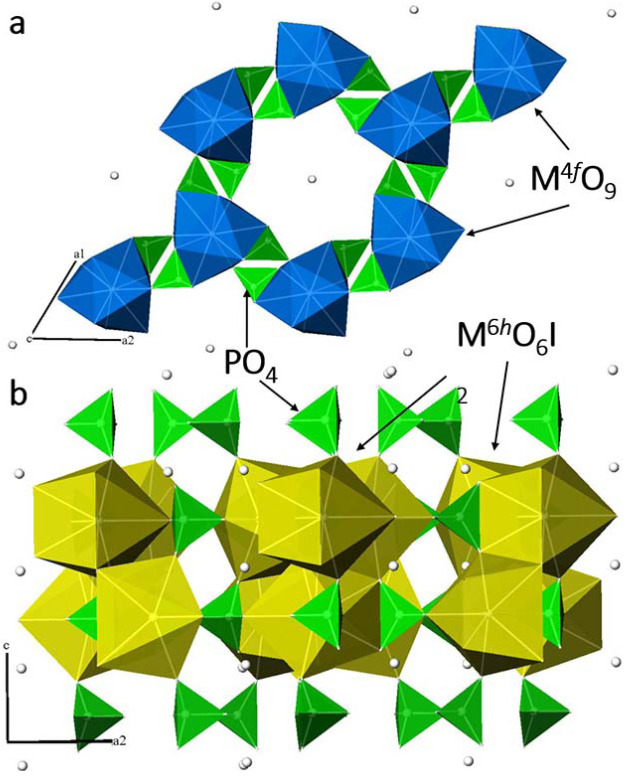
Schematic representation of the crystal structure of the obtained iodine apatites. All positions of iodine atoms are indicated by white spheres in the figure.

As you can see from the Table 4, all three obtained apatites are characterized by a high defectiveness of the positions occupied by halogen, which indicates the low efficiency of the structural type of apatite in relation to the binding of iodine ions, despite the rather optimistic forecast in [15]. In this case, iodine ions are located in the crystallographic position 2*b* (0; 0; 0), which is located on one side in the hexahedral tunnel of the structure formed by three-cap trigonal prisms M^4^ *^f^* O_9_ (Figure 2a), on the other hand, between quasi-layers formed by phosphate tetrahedra and polyhedra M^6^ *^h^* O_6_I_2_ (Figure 2b), which is typical for halogens larger than fluorine in the apatite structure [25–27].

**Table 4:** Atomic coordinates and occupancies of positions of synthesized iodine apatites.

Atom	Wycoff position	x	y	z	Occ
BaPI					
Ba1	4f	1/3	2/3	-0.0001(13)	0.8871(43)
Ba2	6h	0.24113(50)	–0.01859(52)	1/4	0.9152(29)
P	6h	0.3974(19)	0.3632(21)	1/4	1
O1	6h	0.3446(40)	0.4772(35)	1/4	1
O2	6h	0.5808(38)	0.4778(36)	1/4	1
O3	12i	0.3579(19)	0.2752(20)	0.0698(25)	1
I	2b	0	0	0	0.040(24)
PbPI(ss)					
Pb1	4f	1/3	2/3	0.0020(14)	0.9090(22)
Pb2	6h	0.24605(35)	–0.00024(57)	1/4	0.9152(16)
P	6h	0.3911(12)	0.3603(15)	1/4	1
O1	6h	0.3446(28)	0.5118(24)	1/4	1
O2	6h	0.5792(26)	0.4796(28)	1/4	1
O3	12i	0.3508(15)	0.2670(17)	0.0716(18)	1
I	2b	0	0	0	0.127(13)
PbPI(w)					
Pb1	4f	1/3	2/3	0.01250(74)	0.9498(30)
Pb2	6h	0.24646(37)	–0.00620(59)	1/4	0.8940(21)
P	6h	0.3859(16)	0.3412(21)	1/4	1
O1	6h	0.3942(38)	0.4796(26)	1/4	1
O2	6h	0.6095(44)	0.4803(33)	1/4	1
O3	12i	0.3337(17)	0.2403(18)	0.0672(24)	1
I	2b	0	0	0	0.163(18)

**Figure 3:**
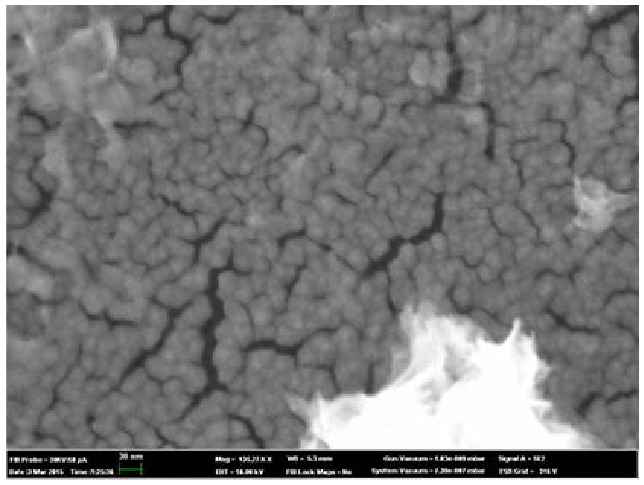
AFM image of PbPI(w) nanospheres. The average particle size is 25–30 nm.

The significantly broader diffraction maxima of PbPI (w) as compared to other obtained apatites indicate the nanoscale of the sample particles. According to calculations using the Scherrer formula and when refining the structure using the Rietveld method, the particle size is 19.6 and 27.6 nm, respectively. The particle size was also confirmed by a direct method - atomic scanning microscopy (Figure 3).

**Figure 4:**
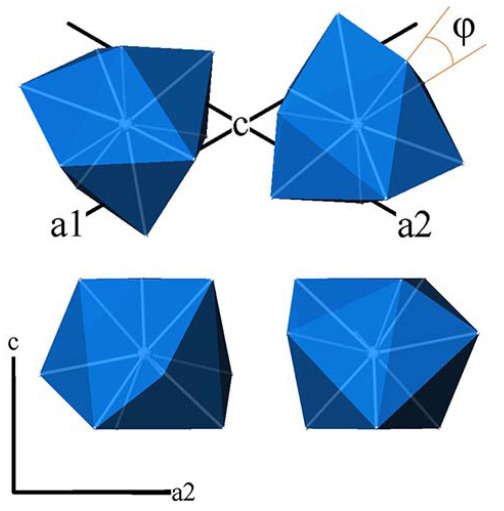
General view of M4fO9 polyhedra in projections onto different crystallographic planes and the “twist angle” φ, which serves as a criterion for the distortion of the crystal structure of apatites.

The abnormal values of the crystal structure distortion index of the obtained apatites are presumably related to the particle size. In a number of works by T. White and colleagues [7, 28], the angle *φ* is the angle of rotation of the bases of three-point trigonal prisms M^4^ *^f^* O_9_ relative to each other - is considered as such an indicator (Figure 4). As you can see from the Table 5, the value of this index for the PbPI (w) sample is as close to 0° as possible, while BaPI and PbPI (ss) have the values of the angle *φ* typical for apatites with large divalent cations [7].

**Table 5:** Parameters of the coordination polyhedrons of cations in the crystal structure of the synthesized iodine-apatites.

	BaPI	PbPI(ss)	PbPI(w)
М4f-O1×3	2.771(30)	2.433(20)	2.651(30)
М4f-O2×3	2.817(33)	2.755(25)	2.824(28)
М4f-O3×3	2.944(21)	2.902(15)	2.996(17)
φ (deg)	18.8	18.3	2.1
			
М6h-O2	2.487(31)	2.376(22)	2.335(28)
М6h-O3×2	2.618(19)	2.571(14)	2.494(18)
М6h-O1	2.929(32)	3.087(21)	2.531(18)
М6h-O3×2	2.956(19)	2.658(16)	3.168(27)
М6h-I×2	3.190(18)	3.060(26)	3.083(12)
			
P-O1	1.504(45)	1.628(23)	1.327(37)
P-O3×2	1.593(21)	1.549(15)	1.607(20)
P-O2	1.634(31)	1.772(34)	1.930(37)

**Figure 5:**
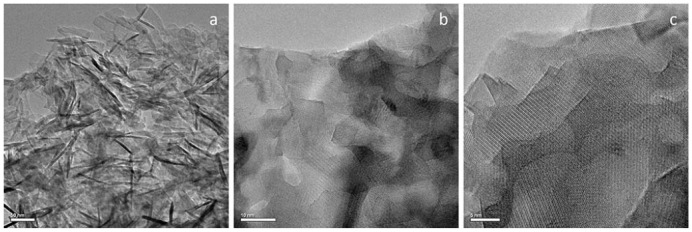
HRTEM image of the microstructure of a PbPI (ss) sample: a - general view of whiskers, b, c - atomic structure of whiskers.

It should also be noted that, according to high-resolution transmission electron microscopy data, during the synthesis of PbPI (ss), whiskers were formed in the sample - one-dimensional dislocation-free crystals 50–100 nm long and 5–10 nm wide (Figure 5a). Figures 5b and 5c also show the crystal structures of agglomerated whiskers. Such whiskers can play the role of a native reinforcing agent when creating a ceramic material based on this compound.

## 4. Conclusion

Despite the theoretical prediction of the possibility of obtaining various iodine-apatites, in particular, only one iodide triphosphates, solution and solid-phase methods were able to obtain two individual compounds, the compositions of which can be described by the following formulas Ba^4^ *^f^*_1.78(2)_Ba^6^ *^h^*_2.75(2)_(PO_4_)_3_I_0.04(2)_, Pb^4^ *^f^*_1.82(2)_Pb^6^ *^h^*_2.75(2)_(PO_4_)_3_I_0.13(2)_ (ss), Pb^4^ *^f^*_1.90(2)_Pb^6^ *^h^*_2.68(2)_(PO_4_)_3_I_0.16(2)_ (w), and pentalead iodide triphosphate was not considered as possible. Despite the low iodine content in the obtained phases, such nonstoichiometric compounds, nevertheless, proved to be quite stable, but only for the largest cations of the studied series, barium and lead. This can be explained by the fact that iodine cations are located between the layers of the structure formed by phosphate tetrahedra, while the interlayer distance is precisely determined by the size of the cation at the 4*f* crystallographic position. In addition, phosphate phases with completely vacant halogen positions (for example, Pb_9_(PO)_6_□ [29]) are known, which have an apatite structure despite the absence of a halogen. This can be attributed to the stability of the obtained phases, which have a largely similar composition and structure and differ only in the presence of a small amount of halogen and an additional amount of cation necessary to maintain electroneutrality.

An analysis of the crystal structure of the obtained compounds showed that these phases bind about 80% less iodine ions from the theoretically expected amount, which cannot speak in their favor as a promising basis for creating a matrix for binding radioactive iodine. However, it should be noted that, during the solid-phase synthesis of PbPI-apatite, nanowhiskers are formed directly in the polycrystalline sample, which can have a favorable effect on the strength characteristics of ceramic materials based on it. Moreover, phosphates with the apatite structure are characterized by higher melting points and polymorphic transformations [30], which makes them more preferable as a chemical base of ceramics for binding radioactive isotopes of iodine.
